# The effects of an immersive virtual reality and telemedicine-based multi-component intervention in individuals with subjective cognitive decline: study protocol of a randomized controlled trial

**DOI:** 10.3389/fpsyg.2025.1591239

**Published:** 2025-07-11

**Authors:** Maria Stefania De Simone, Silvia Zabberoni, Alberto Costa, Erika Tenaglia, Gaetano Tieri

**Affiliations:** ^1^Department of Economics, Psychology, Communication, Education, and Motor Sciences, Niccolò Cusano University, Rome, Italy; ^2^Department of Clinical Neuroscience and Neurorehabilitation, Laboratory of Neuropsychology of Memory, IRCCS Santa Lucia Foundation, Rome, Italy; ^3^Virtual Reality and Digital Neuroscience Lab, Department of Law and Digital Society, University of Rome Unitelma Sapienza, Rome, Italy

**Keywords:** subjective cognitive decline, memory, cognitive training, education program, immersive virtual reality, telemedicine

## Abstract

**Background:**

Older adults with subjective cognitive decline (SCD) are at high risk of developing dementia and frequently experience subclinical symptoms (e.g., anxiety, depression) which are themselves associated with an increased dementia and cognitive decline risk. Our aim is to test the effects of an immersive virtual reality (IVR) and telemedicine-based multi-component intervention, that combines cognitive training and health and lifestyle education program, in individuals with SCD.

**Methods:**

To assess the efficacy of the intervention, a randomized, double-blinded controlled trial will be conducted on a sample of 75 individuals with SCD. Participants will be randomly assigned to one of three conditions: (a) multi-component intervention, including SCD-tailored cognitive IVR training plus a health and lifestyle education program, (b) cognitive-only intervention, including SCD-tailored cognitive IVR training *plus* an active control for the education program, and (c) active control intervention for both cognitive training and education program. All interventions are implemented in IVR environment and will be administered through telemedicine approach. Interventions will be provided in 20 at-home sessions (4 sessions/week, each lasting about 30 min) over a period of 5 consecutive weeks. Outcome measures will be collected before and immediately after intervention. The primary outcome is the effect of the multi-component intervention in enhancing objective cognitive functioning. Secondary outcomes include changes in subjective cognitive functioning, psychiatric symptoms, quality of life and functional neural connectivity. Users' compliance with IVR and telemedicine approach will be also evaluated, as well as individuals' factors affecting training efficacy.

**Discussion:**

Our results will provide new evidence of the efficacy of an innovative multi-component intervention integrating IVR and telemedicine in SCD individuals. Considering the relatively low costs and easy accessibility, it could be a valuable contribution to primary prevention initiatives for dementia risk reduction.

**Trial registration:**

This trial was prospectively registered at ClinicalTrials.gov on May 24, 2024 (registration number: NCT06429215).

## 1 Introduction

Dementia and cognitive decline represent a significant and growing global health crisis. The World Alzheimer Report 2024 estimated 55 million people living with dementia worldwide in 2019, with Alzheimer's disease (AD) being the most prevalent cause. This number is projected to nearly double every 20 years, reaching approximately 139 million by 2050, primarily due to population aging (“Aging and Health,” World Health Organization). Dementia and cognitive decline are leading contributors to disability and dependency among older adults, incurring an estimated cost of US$1.3 trillion in 2019 (Wimo et al., 2023); this leads to a huge burden on society and healthcare system that is destined to increase gradually over time. Despite considerable research efforts have been made to find effective disease-modifying treatments, to date there is no intervention known to cure or even reliably affect the course of dementia (Imbimbo et al., 2020). Consequently, there is increasing recognition of the need to move away from an exclusive focus on treatment of dementia toward the development of early effective prevention-interventions aimed at preventing or delaying the progression to cognitive impairment and dementia in aging population (World Health Organization, 2024). In fact, it has been estimated that up to 40% of dementia cases could potentially be prevented by addressing modifiable risk factors, including cognitive activity, diet, physical exercise, and psychiatric conditions like anxiety, apathy, and depression (Livingston et al., 2020).

Within this framework, cognitive training has garnered significant attention as a promising non-pharmacological intervention for improving cognition and potentially delaying clinical progression, whose beneficial effects are likely mediated by brain plasticity and increased cognitive reserve (Greenwood and Parasuraman, 2010). Importantly, even in older adults, synaptic structures retain modifiability, making them amenable to brain plasticity induced by cognitive training (Yin et al., 2022). Even relatively short-duration cognitive training sessions (4–8 weeks) have, in fact, demonstrated significant effects on functional connectivity in clinically healthy elderly (Kang et al., 2021; De Simone et al., 2023a; Cotelli et al., 2019; Costanzo et al., 2020; Rolandi et al., 2025). Because preventative interventions are most effective before the onset of clinically significant symptoms, subjective cognitive decline (SCD) is a crucial target for acting early interventions aimed at delaying or modifying the disease course. SCD, which is characterized by self-perceived cognitive decline without objective impairment on standard neuropsychological tests (Jessen et al., 2020), represents a high-risk factor for accelerated cognitive decline and the development of mild cognitive impairment and dementia, particularly when accompanied by concern (Jessen et al., 2020). In a certain proportion of individuals, SCD is considered the earliest preclinical indicator of AD, in that it is associated with increased likelihood of positive biomarkers and neurodegeneration consistent with AD pathology; for these cases, targeting preventive treatments at this stage holds the potential to slow or even prevent progression to dementia. Beyond its association with preclinical AD, there is another aspect of treating individuals with SCD, which is related to distressing symptoms that they experience. The self-perception of cognitive decline often leads to increased stress and fear of dementia, which in turn contribute to psychiatric symptoms like depression, anxiety, and apathy, generally and specifically related to subjectively perceived cognitive deterioration (Jessen et al., 2020). For instance, it has been demonstrated that concern about loss of cognitive function contributes to elevated anxiety observed in individuals with SCD, and anxiety in turn increases alertness for cognitive failures (Hill et al., 2016). As a result, SCD has also been associated with lower quality of life, reduced mental wellbeing perception and worsening of positive psychology constructs such as self-esteem and self-efficacy (Roehr et al., 2017; Su et al., 2023). All these factors, in turn, have a negative impact on brain structure and function, and increase the risk of cognitive decline and dementia. Therefore, early and effective preventive interventions could not only benefit the mental health of individuals with SCD but also potentially reduce the risk of future clinical progression.

Non-pharmacological interventions for SCD have received increasing attention in recent years. A meta-analysis by Sheng et al. (2020) of 18 randomized controlled trials investigating interventions for SCD (including psychological and health education, nutritional supplementation, cognitive training, and multi-domain interventions) found that cognitive training had a small effect on objective memory and significant improvements in subjective memory and psychological wellbeing. While some recent studies have corroborated the positive effects of cognitive training on cognitive function (Yu et al., 2024), others have reported conflicting results (Cheng et al., 2018; Hong et al., 2020; Bhome et al., 2018). Given the current lack of definitive evidence to guide recommendations and implement clinically practical interventions, further research is urgently needed.

Building upon recent advancements in computer technology, the emergence of new generations of Immersive Virtual Reality (IVR) has unlocked considerable potential for novel clinical applications (Tieri et al., 2018). These systems are particularly promising due to their capacity to offer greater ecological validity, allowing users to interact with virtual stimuli in a naturalistic manner using their own body movements, to experience the illusory sensation of “being physically present” in the virtual environment (i.e., sense of presence; Sanchez-Vives and Slater, 2005), and to enhance patient compliance (Tieri et al., 2018; Sanchez-Vives and Slater, 2005; Fusco and Tieri, 2022).

In light of the foregoing, this study aims to evaluate the effectiveness of an immersive virtual-reality (IVR) and telemedicine-based multi-component intervention, combining cognitive training and a health and lifestyle education program, on objective and subjective cognitive function, psychiatric symptoms, quality of life and brain connectivity in older adults at high-risk for cognitive decline and dementia (i.e., SCD). The primary objective is to determine whether the multi-component intervention, when compared to an active control condition, leads to significant post-intervention gains in objective cognitive functioning, and whether these gains are greater than those induced by a cognitive-only condition. The secondary objectives are: (1) to assess the transfer effect of the intervention on self-perceived cognitive functioning, psychiatric symptoms and quality of life; (2) to assess intervention-induced changes in whole-brain functional connectivity, as highlighted by resting state-fMRI images; (3) to assess user compliance with telemedicine approach combined with IVR. Exploratory objectives include: (1) investigating potential moderators of intervention efficacy (e.g., demographics, cognitive reserve, SCD severity, lifestyle and dementia risk factors); and (2) evaluating hypotheses regarding neuroimaging results and their association with behavioral data (e.g., correlation between behavioral and imaging data, association between post-training brain changes and participants' characteristics, and whether intervention-induced improvements rely on specialized brain areas or depend on compensatory responses from other regions).

## 2 Methods/design

### 2.1 Study design

This 5-week randomized controlled trial (RCT) use a double-blinded, three-parallel group design, guided in accordance with the Consolidated Standards of Reporting Trials (CONSORT) guidelines (Bennett, 2005). Following a screening phase to verify eligibility criteria through a comprehensive clinical and neuropsychological evaluation, and after obtaining informed consent, enrolled participants with SCD will undergo an in-person baseline assessment and a 3T MRI scan (where feasible) to collect clinical, neuropsychological, behavioral, and neuroimaging data prior to the intervention (T0). Then, using a stratified randomization method (ratio 1:1:1, taking age into account), participants will be allocated in a blinded fashion to one of the three experimental conditions:

(1) a multi-component intervention (MC-I), including SCD-tailored cognitive IVR training *plus* a health and lifestyle education program;
(2) a cognitive-only intervention (CO-I), including SCD-tailored cognitive IVR training *plus* an active control for the education program;
(3) an active control intervention (AC-I), including active controls for both cognitive training and education program.

The active control for the cognitive training (in the AC-I condition) involves IVR tasks mirroring the experimental training's structure but with very low cognitive demands. The active control for the education program (in both CO-I and AC-I conditions) consists of educational videos on topics unrelated to dementia and cognitive decline prevention. A detailed description of the interventions is provided in paragraph 2.7.

**Figure 1:**
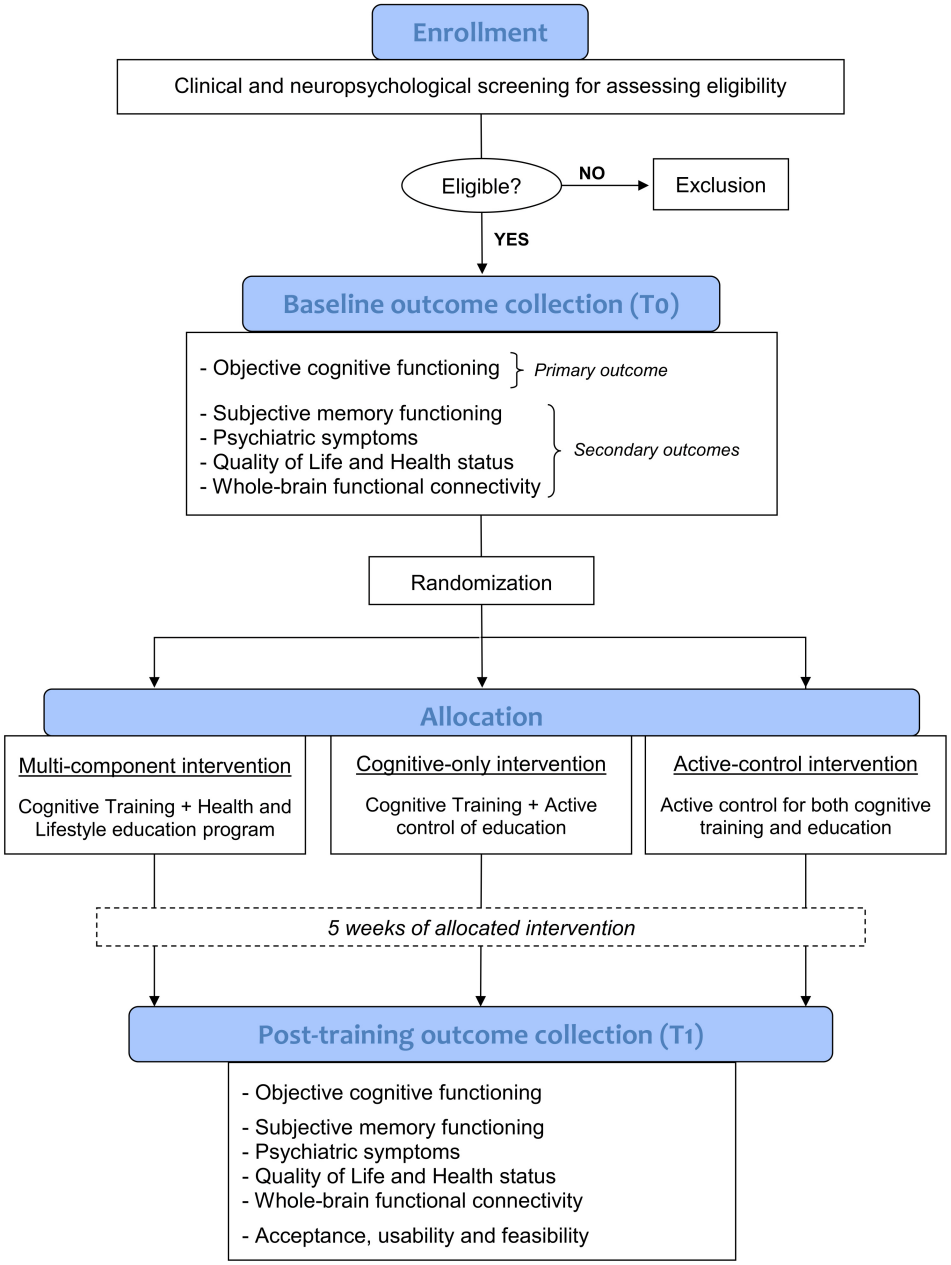
Flow diagram of the study design.

All three groups will receive 20 training sessions (4 sessions per week, each lasting about 30 min) over a period of 5 consecutive weeks. Participants will complete their respective interventions at home using virtual reality head-mounted displays (HMD) remotely controlled and monitored in real-time via a dedicated telemedicine platform. Prior to the intervention, all participants will receive in-person training on the use of HMD tool. Within 48 h and 1 week of completing the 5-week intervention, participants will return respectively for a post-intervention 3T MRI scan (where feasible) and an in-person evaluation, in order to collect post-training clinical, behavioral, neuropsychological and neuroimaging outcome measures (T1). A study flowchart is provided in Figure 1.

### 2.2 Participants

A sample of 75 individuals who refer to the Center for Cognitive Disorders and Dementias (CDCD) of IRCCS Santa Lucia Foundation because of a self-report of cognitive impairments and who meet the current criteria for SCD (Jessen et al., 2020) will be recruited. We are focusing on SCD subjects to be recruited from a memory clinic in accordance with evidence that help-seeking behavior in SCD has been related to a higher risk of progressive cognitive decline and dementia, as well as underlying AD pathology (Jessen et al., 2020). SCD will be defined in agreement with recently proposed recommendations (Jessen et al., 2020). Specifically, inclusion criteria are: (a) over 55 years of age; (b) absence of neurological or psychiatric disorders; (c) no history of alcohol or drug abuse; (d) Mini-Mental State Examination (MMSE) score ≥28; (e) age-appropriate cognitive functioning as confirmed by performance above the normality cut-off scores on all tests comprising the extensive neuropsychological battery administered during the screening phase (for details, see “*Neuropsychological screening battery”* section); (f) concern over a self-experienced cognitive decline. The latter will be operationalized with the following widely-used procedure (De Simone et al., 2023b). First, we ask participants “Do you feel like your cognitive ability (e.g., memory, concentration, planning, orientation, or language) has become worse?” (possible answers: yes/no). In case of a positive response we ask whether the perceived decline is experienced as worrisome by asking “Does this worry you?” (possible answers: yes/no). SCD will be defined by endorsement of perceived decline with concern about cognitive decline.

Additional inclusion criteria are ability to give their written consent to participate in the study, normal or corrected to normal vision and hearing, native or fluent proficiency in Italian, ability to commit to the whole intervention and follow-up assessment.

This RCT study is being conducted in accordance with the Declaration of Helsinki. The study protocol has been approved by the Territorial Ethics Committee of Lazio Area 5 (experiment register N.81/SL/23) and has been prospectively registered at ClinicalTrials.gov on May 24, 2024 (registration number: NCT06429215). Any significant changes in the protocol will be reported in the trial registry. Written informed consent will be obtained from each participant.

### 2.3 Sample size calculation

To estimate the appropriate sample size, the G^*^Power program was used. Considering an effect size of 0.37 obtained in a recent meta-analysis on cognitive intervention in SCD (Sheng et al., 2020), a power of 0.8 and an alpha of *p* < 0.05, we applied repeated measurements of multivariate analysis of variance (three groups: MC-I group, CO-I group, AC-I group; two measurements: pre-intervention and post-intervention) for analysis. The appropriate sample size was determined to be 74. Therefore, each of the three experimental groups requires at least 25 participants.

### 2.4 Neuropsychological screening battery

The tests comprising the standardized neuropsychological battery that is administered in the screening phase to assess inclusion criteria are described below according to the cognitive domains they examine:

(a) Global cognitive functioning: mini mental state examination (MMSE) (Magni et al., 1996);
(b) Verbal episodic long-term memory: 15-word list test (immediate, 15-min delayed recall and 30-min recognition trial) and short story test (immediate and 20-min delayed recall; Carlesimo et al., 1996, 2002);
(c) Short-term memory and working memory: digit span test forward and backward (Monaco et al., 2013);
(d) Attention and Executive functions: phonological word fluency (Carlesimo et al., 1996), semantic word fluency test (Costa et al., 2014), modified card sorting test (Nocentini et al., 2002), trail making test parts A and B (Giovagnoli et al., 1996);
(e) Reasoning: Raven's colored progressive matrices (Carlesimo et al., 1996);
(f) Constructional praxis: copy of simple drawings and copy of drawing with landmarks (Carlesimo et al., 1996);
(g) Language: naming subtest of the ENPA (NeuroPsychological Examination for Aphasia; Capasso and Miceli, 2001).

For all tests but one (i.e., ENPA naming) we use Italian normative data for score adjustment (sex, age, and education) and to define normality cut-off scores, which were established as the lower limit of the 95% tolerance interval for a confidence level of 95%.

### 2.5 Outcome measures

Outcome measures will be collected pre-intervention (T0) and immediately after the 5-week intervention period (T1). The primary outcome is the effect of the intervention on objective cognitive functioning. Previous studies on non-pharmacological intervention in SCD generally included standard neuropsychological tests as cognitive outcomes to evaluate post-training changes (e.g., MMSE, ADAS-Cog). However, SCD performs normally in standard neuropsychological tests and typically exhibits both ceiling and practice effects on consecutive evaluations with traditional tasks (Campos-Magdaleno et al., 2017; De Simone et al., 2021). Thus, it is necessary to adopt more-challenging cognitive outcomes for ascertaining treatment efficacy. Novel paradigms that are sensitive enough to objectively detect the subtle cognitive decline complained by SCD have been recently proposed (De Simone et al., 2023b; Kormas et al., 2020; Markova et al., 2023). In particular, in a recent paper of our group, we demonstrated that tasks assessing specific cognitive processes (i.e., associative long-term memory, working memory binding, spatial pattern separation) significantly differentiate SCD individuals from healthy controls without SCD with good overall accuracy (De Simone et al., 2023b). In addition, these tests were significantly associated with self-perceived memory functioning. According to these results, intervention-induced changes in cognition will be assessed in this study through the following tasks: (a) Face–Name Associative Memory Exam—short form (FNAME-12; De Simone et al., 2023b; Papp et al., 2014), which evaluates long-term associative memory ability; (b) Visual Short-term Memory Binding test (Parra et al., 2019), which evaluates conjunctive binding ability; (c) Spatial pattern separation test (De Simone et al., 2023b; Holden et al., 2012), which evaluates pattern separation processes; (d) Semantic-phonemic verbal fluency discrepancy (Vaughan et al., 2018). For FNAME-12, a parallel form (i.e., alternative version using similar material) will be applied for T1 evaluation in order to reduce the effects of learning and practice.

Secondary outcomes include the evaluation of intervention-induced changes in: (a) Subjective memory functioning, using Subjective Memory Complaints Questionnaire (De Simone et al., 2023b) and Multifactorial Memory Questionnaire (Raimo et al., 2016); (b) Psychiatric symptoms, using Beck Depression Inventory (BDI; Beck et al., 1961) and State–Trait Anxiety Inventory (STAI; Spielberger, 1983); (c) Quality of Life and Health status, using Short Form-36 Health Survey (Apolone and Mosconi, 1998) and Patient Global Impression of Change—PGIC (Hurst and Bolton, 2004); (d) Whole-brain functional connectivity.

Additionally, participants will be asked to complete the following questionnaires during the post-training evaluation (T1) in order to assess the acceptance, usability, and feasibility of the proposed IVR and telemedicine-based program: Simulator Sickness Questionnaire (Kennedy et al., 1993), User Satisfaction Evaluation Questionnaire (Gil-Gómez et al., 2017), Nasa Task Load Index (Hart and Staveland, 1988), User Experience Questionnaire (Laugwitz et al., 2008) and a modified version of the System Usability Scale (Brooke, 1995; Grier et al., 2013) that also includes an open question for qualitative input about users' experience.

For further exploratory analyses, we will additionally collect measures of cognitive reserve by means of the Cognitive Reserve Index questionnaire (Nucci et al., 2012) and dementia risk factors by adapting in Italian several items of the Lifestyle for Brain Health (Schiepers et al., 2018), in order to evaluate the potential impact of underlying mechanisms on the efficacy of intervention. Data related to participants' performance on the IVR cognitive tasks (e.g., accuracy, timing), which will be collected through the telemedicine platform for each individual at-home training session, will be also considered for analyses.

### 2.6 Prior to intervention

Prior to beginning the at-home intervention, all participants will be trained in the use of the IVR HMD tool in an in-person session. During the in-person onboarding session, an experimenter individually guides each participant through the main features and functionalities of the HMD tool. This includes demonstrating how to power the device on/off, recharge it, connect to Wi-Fi, calibrate the virtual environment, and access the intervention. Crucially, the experimenter also shows participants the exercises to perform (according to allocation) through video tutorials and has each participant complete a trial level directly with the HMD to ensure they are comfortable and proficient before starting the training independently. This hands-on, individualized guidance is essential to ensure that participants, many of whom may be new to VR technology, are fully confident and competent in using the device and accessing the training safely and effectively from home. Moreover, the participants are given an ad hoc user's manual in paper format that provides detailed information about the HMD operating mode and the activities to be carried out. In this preliminary session, the experimenter also makes agreements with each participant regarding the specific days on which the exercises should be performed during the 5 weeks of intervention. At the end of the session, a dedicated IVR HMD is delivered to each participant to perform the 5-week intervention at home. Throughout the intervention period, remote technical support is available upon request. Interventions are scheduled daily and continuously monitored through the telemedicine platform. The platform provides real-time tracking of whether participants have initiated and completed their assigned sessions. Should a participant miss a session, the system automatically notifies the experimenter, prompting proactive contact to reschedule the session for the immediate following day. This approach ensures that the prescribed frequency and dose of the intervention are maintained as closely as possible. However, if three consecutive exercise sessions are missed despite the experimenter's prompts, the participant's intervention is terminated.

### 2.7 Intervention

All interventions (i.e., MC-I, CO-I, AC-I) are implemented in IVR environment and will be administered and managed through telemedicine approach.

#### 2.7.1 Multi-component intervention

**Figure 2:**
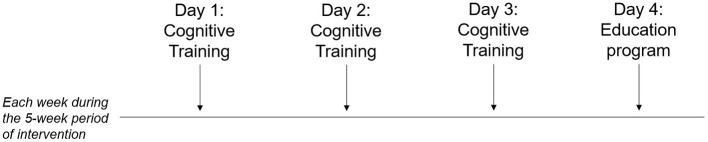
Temporal organization of the Multi-component intervention over the 5-week period.

Multi-component intervention (MC-I) includes both a SCD-tailored cognitive training (3 sessions/week, each lasting about 30 min) and a health and lifestyle education program (1 session/week, each lasting about 20 min), for a total of 20 sessions during a consecutive 5-week period (Figure 2).

##### 2.7.1.1 Cognitive training

The selection of the most appropriate cognitive activities to be trained was made on the basis of empirical findings showing that individuals with SCD, while performing normally on traditional neuropsychological tests, have objective deficits in specific and more demanding cognitive processes (e.g., long-term associative memory, short-term memory binding, spatial pattern separation) when compared with healthy individuals without SCD (De Simone et al., 2023b; Kormas et al., 2020; Markova et al., 2023). For example, difficulty remembering face-name associations is one of the most commonly reported memory difficulties in SCD that underlines a subtle deterioration of associative memory processes, as demonstrated in our recent paper (De Simone et al., 2023b). As such, training to enhance memory for face-name associations could be an important priority for ecologically relevant improvement in SCD. Accordingly, our IVR cognitive intervention was developed to enhance those specific processes that have been consistently found to be impaired in SCD. In particular, two different tasks were implemented in virtual real-like scenarios of daily living situations, which target the following cognitive processes, respectively: (1) long-term associative memory (i.e., the ability to learn and remember associations between previously unrelated items), (2) working memory conjunctive and relational binding (i.e., the ability to integrate multiple features within an object representation or to associate one item to its spatial or temporal context), and (3) spatial pattern separation (i.e. the ability to differentiate partially overlapping patterns of activation in order to retrieve one pattern as separate from others that are similar).

**Figure 3:**
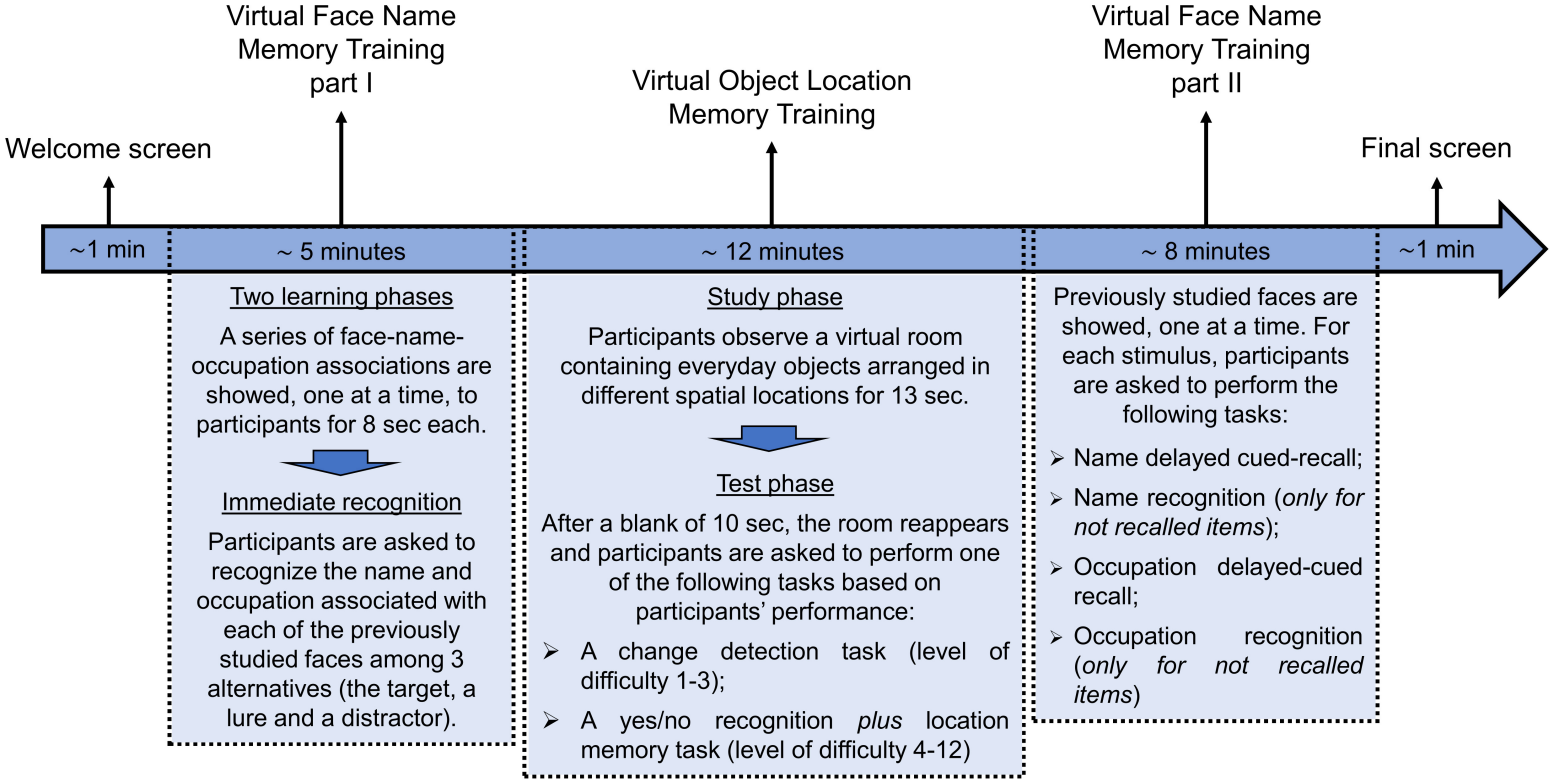
Temporal organization of an individual session of cognitive training.

**Figure 4:**
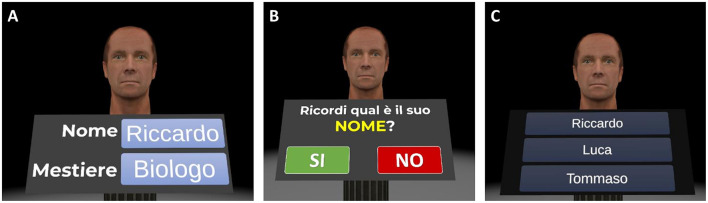
Virtual face-name memory training—part I. Example of the virtual face-name memory training—part I. **(A)** During the two learning phases, a series of 3D faces, each associated with a name and an occupation (e.g., name: Riccardo; occupation: biologist), are displayed for 8 s in sequence. Then **(B)** participants are shown the previously learned faces, one at a time, and **(C)** asked to perform a three-alternative forced-choice recognition to identify the correct name and occupation (Translation of the instructions in **(B)** “*Do you remember his name*?”).

The temporal sequence of the activity to performed in each individual session of cognitive training is represented in Figure 3 and described in detail below:

1) *Welcome screen (about 1 min)*. Participants are greeted with a customized welcome screen before beginning the tasks. Participants are asked to rate their current state using visual analog scales (VAS) ranging from 0 (*not at all*) to 100 (*a lot*) for three questions: “*Do you feel good right now?,” “Do you feel motivated?,”* and “*Are you tired?.”* Participants then click the start button to begin the cognitive exercises.
2) *Virtual Face Name Memory training—Part I (about 5 min)*. This first part of the virtual face-name memory training consists of two learning phases followed by an immediate names and occupations recognition test. At the beginning of the task, a series of 3D faces, each associated with a name and an occupation, are presented individually for 8 s (Figure 4A). Participants are asked to read aloud the name and occupation associated with each face in order to ensure that they are attending to the items. At the end of the series, this procedure is repeated for the second learning phase: the same set of face-name-occupation associations are presented again (in a different order) and participants are asked to read aloud the name and occupation for each face. Following the second learning phase, participants are shown the previously learned faces, one at a time, and asked to perform a three-alternative forced-choice recognition, selecting the correct name and occupation (target) from a lure (a name/occupation paired with a different face) and a distractor (a novel, unstudied name/occupation; Figures 4B, C).

Faces are balanced for sex (females and males) and age (young adults and seniors) within each training session. The number of face-name-occupation associations to be learned and subsequently recalled progressively increases based on the participant's performance. All participants start with 5 face-name-occupation associations in the first training session (Day 1). The number of associations increases by one in subsequent sessions, up to a maximum of 20, after achieving at least 70% accuracy in the name delayed cued-recall task described in the “*Virtual Face Name Memory training—Part II”* section.

**Figure 5:**
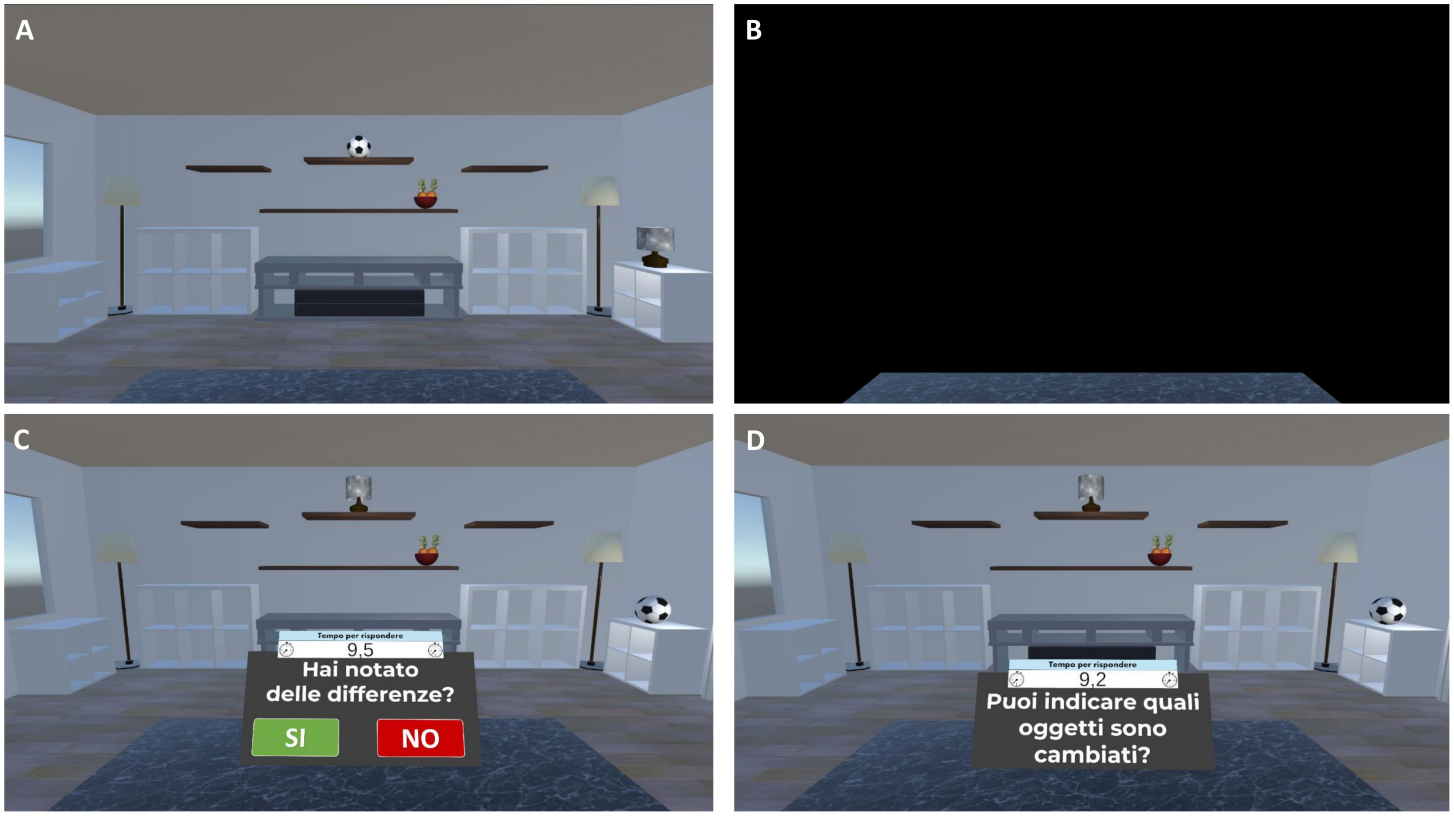
Virtual object-location memory training—change detection task. Example of the virtual object-location memory training—change detection task. **(A)** Participants are presented with a virtual living room containing 3D objects in specific spatial configurations for 13 s. **(B)** The scene is then occluded by a black panel for 10 s. **(C)** Subsequently, the living room reappears, either with the same or a modified object-location arrangement. Participants respond “Yes” or “No” to indicate whether any changes were detected (Translation of the instructions in **(C)** “*Have you noticed any differences?*”). **(D)** If a change is correctly identified, participants are prompted to select the altered objects or locations (Translation of the instructions in **(D)** “*Can you indicate which items have changed?*”).

**Figure 6:**
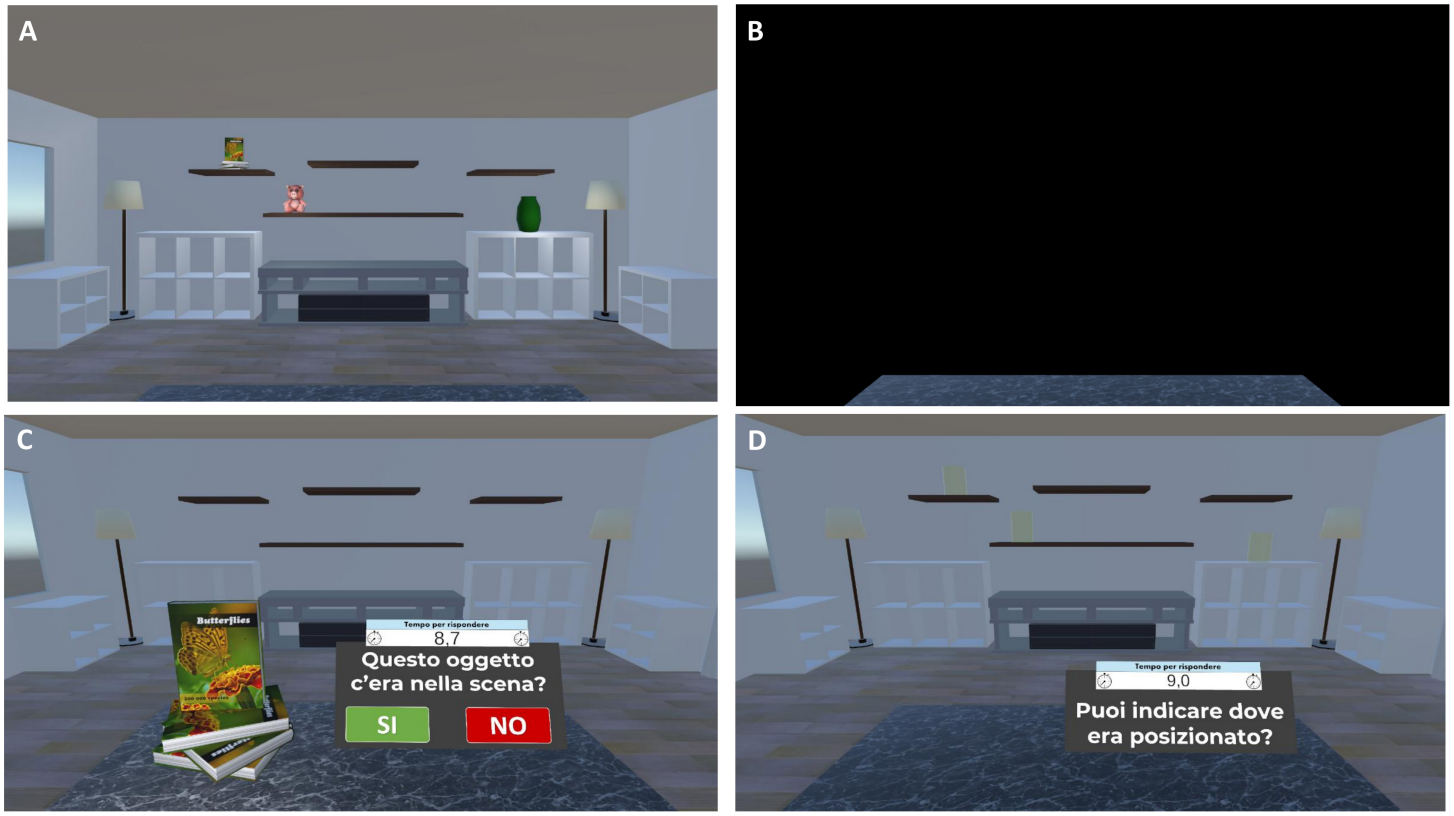
Virtual object-location memory training—yes/no recognition *plus* location memory task. Example of the virtual object-location memory training—yes/no recognition plus location memory task. **(A)** Participants are presented with a virtual living room containing 3D objects in specific spatial configurations for 13 s. **(B)** The scene is then occluded by a black panel for 10 s. **(C)** Subsequently, individual 3D objects are presented and participants are required to perform a yes/no recognition, indicating whether the presented item is a studied or an unstudied object (Translation of the instructions in **(C)** “*Was this object present in the scene?*”). **(D)** If the object is correctly recognized, participants are prompted to localize it by clicking on the corresponding yellow cylinder representing its original location (Translation of the instructions in **(D)** “*Can you indicate where it was located?*”).

3) *Virtual Object Location Memory training (about 12 min)*. Virtual object location memory training consists of two tasks of increasing difficulty: a change-detection task (levels of difficulty from 1 to 3) and a yes/no recognition *plus* location memory task (levels of difficulty from 4 to 12). In both tasks, the scenario consists of a room containing several everyday objects in different spatial locations. During the study phase, participants are asked to carefully observe the scene at 180° and memorize the objects and their spatial locations. After a 13-s exposure, the room disappears. Following a 10-s blank (retention interval), the test phase is administered in change detection or yes/no recognition test format. Specifically:

- For the change-detection task, the room reappears with: (1) same objects in the same positions (no-change condition), (2) same objects in different positions (position-change condition), or (3) different objects in the same positions (object-change condition). Participants are asked to respond Yes or No to indicate whether the items and their locations in the study and test phases are the same or different. Participants who correctly identify a change are asked to click on the altered objects or locations to specify the change (Figure 5);
- For the yes/no recognition *plus* location memory task, the room reappears, and a series of everyday 3D objects are presented individually in front of participants. The series includes items presented in the study phase (targets), new item not previously seen (foils), and new items that are perceptually similar to those seen during the study phase, but not identical (lures). For each stimulus, participants are required first to perform a yes/no recognition, indicating whether the presented item is a studied object (target) or an unstudied one (foil or lure). Then, once an object is correctly recognized as target, participants are asked to drag it to its remembered location (localization memory; Figure 6).

The Virtual Object Location Memory training comprises 12 levels of difficulty, each consisting of six different trials. Task difficulty adaptively increases from Level 1 to Level 12 based on participants' performance. This progression involves varying the task type (change-detection: levels 1–3; yes/no recognition plus location memory: levels 4–12) and the number of objects presented (change-detection: 3–5 items; yes/no recognition plus location memory: 3–11 items). All participants start at the easiest Level 1. After two consecutive successful trials (no errors), they advance to the next level.

**Figure 7:**
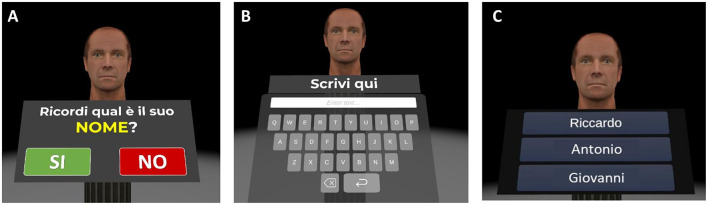
Virtual face-name memory training (part II). Example of the virtual face-name memory training Part II. **(A)** Previously studied faces are presented individually. **(B)** Participants are asked to recall the name associated with each face using a virtual keyboard (name delayed cued-recall). **(C)** For incorrect recalls, participants perform a three-alternative forced-choice recognition task (name recognition).

4) *Virtual Face Name Memory training—Part II (about 8 min)*. The second part of the virtual face-name memory training assesses delayed cued-recall and recognition of the previously studied face-name-occupation associations. Specifically, previously studied faces are presented individually; for each stimulus, participants are asked to perform the following tasks:

(1) Name delayed cued-recall: participants are asked to recall the associated name; responses are provided using a virtual keyboard (Figures 7A, B). (2) Name recognition: for names not correctly recalled, participants perform a three-alternative forced-choice recognition task, selecting the correct name (target) from a novel name (distractor) and a lure (a name paired with a different face; Figure 7C). (3) Occupation Delayed Cued-Recall: participants are asked to recall the associated occupation; responses are provided by using a virtual keyboard. (4) Occupation Recognition: for occupations not correctly recalled, participants perform a three-alternative forced-choice recognition task, selecting the correct occupation (target) from a novel occupation (distractor) and a lure (an occupation paired with a different face).

5) *Final screen (about 1 min)*. The intervention concludes with a final screen. Participants are asked to rate their experience using VAS ranging from 0 (not at all) to 100 (a lot) for three questions: “*Did you experience any discomfort while using the system?,” “How mentally fatiguing were these tasks?,”* and “*How satisfied are you with your performance?.”*

##### 2.7.1.2 Health and lifestyle education program

The health and lifestyle education program consists of five key topics covered in immersive 360° VR videos featuring experts in the field. These videos aim to increase awareness and knowledge of the various health conditions associated with an increased risk of cognitive decline and dementia, empowering participants to develop healthier lifestyles (e.g., encourage healthy dietary habits, incorporate regular physical activity into daily routine, reduce sedentary behavior). The topics that are covered include (1) how aging affects cognition and the brain; (2) modifiable medical risk factors for cognitive decline and dementia, including weight, cholesterol, diabetes, hypertension, and sleep disturbance, as well as modifiable lifestyle risk factors, such as smoking, stress, physical activity, nutrition, and social and cognitive engagement; the protective effect of (3) Mediterranean diet and (4) regular physical and cognitive activities against cognitive impairments and dementia. To induce greater participant engagement, we recorded immersive 360° VR videos with three experts in the field (i.e., a geriatric doctor, a neuropsychologist and a nutritional biologist); the videos were implemented in the HMD. In this way, we could induce sense of presence, giving the participants the illusory sensation to be physically present in the scenario. In addition, to make the educational experience more engaging, interactive questions were implemented into immersive videos.

#### 2.7.2 Cognitive-only intervention

Cognitive-only intervention (CO-I) includes the SCD-tailored cognitive IVR training (3 sessions/week, each lasting about 30 min) and 5 sessions of active control of the education program (1 session/week, each lasting about 20 min). In this latter, participants will view immersive 360° VR educational videos on non-health-related topics (i.e., science and history) and answer specific questions about their content. This serves as a control for the educational component of the MC-I.

#### 2.7.3 Active control intervention

Active control intervention (AC-I) includes an active cognitive control, which serves as a placebo-like condition for the experimental SCD-tailored cognitive IVR training (included in both the MC-I and CO-I interventions). It also incorporates the same educational active control as used in the CO-I intervention.

In the active cognitive control, participants are asked to virtually carry out simple actions within the same virtual environment as those performed in the SCD-tailored cognitive IVR training. However, they have to follow prearranged instructions requiring minimal cognitive processing, typically involving simple step-by-step instructions or repetitive actions. For example, in the *Virtual Face Name Memory* active control, participants are shown the same stimuli (face-name-occupation associations) but are required to execute simple tasks that do not involve memory abilities (e.g., reading aloud the names and occupations associated with the presented faces, judging the pleasantness of faces, judging whether the name is appropriate for the face). Similarly, for the *Virtual Object Location Memory* active control, participants are immersed in the identical scenario as the experimental condition (a room containing several everyday objects in different spatial locations) but are required to perform simple tasks, such as counting the number of objects present in the virtual room. Frequency and duration are identical to those of the CO-I and MC-I conditions.

### 2.8 IVR and TM apparatus

Virtual stimuli, objects and environment were designed using 3DS Max 2022 (Autodesk, Inc.) and Unity 2022.3.5f1 game engine software, respectively. Two distinct virtual environments were developed and implemented. Specifically, for the *Virtual Face Name Memory training*, a minimalist virtual environment was created, characterized by a darkened spatial volume. A single, centrally positioned cylindrical column, illuminated by a focused spotlight, acts as the primary visual element. Facial stimuli, presented sequentially for 8-s intervals, were displayed at the top of the column. The 3D facial models were generated by adapting models of the Microsoft Rocketbox avatar library. For the *Virtual Object Location Memory training*, the virtual environment consists in a large living room with a centrally located table and a series of empty shelving units attached to the front wall. Within these shelves, a varying number of 3D objects are presented based on task difficulty. These 3D objects were designed and optimized using Autodesk 3ds Max 2022.

The tasks were programmed in Unity 2022.3.5f1 and built for the Meta Quest 2 head-mounted display (HMD), with a resolution of 1,832 × 1,920 pixels per eye, a refresh rate of 72 Hz and 6 degrees of freedom (DoF). An intuitive interaction modality was implemented using the Oculus Touch controller, combined with custom Unity C# scripts, enabling ray-cast object selection and trigger-based interaction. Immersive 360° VR videos for *health and lifestyle education program* were recorded using an Insta 360 X4, which captures with 25 fps with 8K resolution in 360° mode.

To remotely control and manage the HMD devices and task sequences, a dedicated telemedicine platform was developed, consisting of a web app developed in C# and implemented with Unity 2022.3.5f1 game engine software. The platform features a dedicated interface allowing the experimenter to remotely schedule daily exercise sessions for each participant, according to their assigned condition. Beyond scheduling, the platform provides real-time tracking of whether participants have initiated and completed their assigned sessions. Should a participant miss a session, the system automatically notifies the experimenter, prompting proactive contact to reschedule the session for the immediate following day. Furthermore, the web app generates detailed performance data from each VR session, offering the experimenter a comprehensive overview through quantitative metrics such as accuracy rates, number of errors, and response times. This integrated system ensures the experimenter can maintain an indirect yet significant control over the participant's experience, thereby guaranteeing adherence to the study protocol and offering valuable quantitative feedback on performance, all while facilitating remote oversight.

### 2.9 Planned statistical analysis

Statistical analyses that will be applied to the whole dataset of outcome measures include descriptive statistics (e.g., means, standard deviations, frequencies) and graphical exploratory data analytic techniques (e.g., residual plots, normal Q-Q plots) that will be performed to describe distributions of the data, to identify potential outliers and missing values, and to check for the possible violation of assumptions necessary for the planned statistical methods. Between-group differences in demographic (i.e., age, education) and screening neuropsychological test performance will be assessed using parametric (i.e., one-way analysis of variance—ANOVA) or non-parametric tests (i.e., Kruskal-Wallis) when appropriate. Chi-square test will be used to assess differences in sex distribution among groups. If data will be of normal distribution, possible between-group differences (MC-I group vs. CO-I group vs. AC-I group) in outcome measures at T0 and T1 will be analyzed using either repeated measures ANOVA or a similar repeated measures statistical technique such as linear mixed models. If linear model assumptions will be severely violated, an analogous non-linear repeated measures statistical approach will be used. In this case, variable transformation may be performed, if appropriate.

#### 2.9.1 MRI acquisition and planned analysis

MRI scans will be acquired using a 3.0T MRI scanner (MAGNETOM Prisma MRI scanner, Siemens Healthcare, Erlangen, Germany) equipped with a 64-channel head-and-neck coil. The MRI protocol includes the following sequences: (a) T2-weighted turbo spin-echo (TSE) [matrix 448 × 448, FOV 220 × 220 mm^2^, 29 transversal slices, slice thickness 4 mm, repetition time (TR) = 3,490 ms, echo time (TE) = 95 ms, acquisition time 2 min 42 s], during which participants will be asked to keep their eyes closed, not to think about anything in particular and not to fall asleep; (b) a fast fluid-attenuated inversion recovery (FLAIR) [matrix 240 × 256, FOV 240 × 256 mm^2^, 176 sagittal slices, slice thickness 1 mm, TR = 8,000 ms, TE = 314 ms, inversion time (TI) = 2,350 ms, acquisition time 7 min 54 s]; (c) T1-weighted multi-echo MPRAGE sequence (MEMPRAGE) [matrix 256 × 256, FOV 256 × 256 mm^2^, 176 sagittal slices, slice thickness 1 mm, TR = 2,500 ms, TE = 1.67/3.48/5.29/7.10 ms, TI = 1,080 ms, flip angle 8°, acquisition time 7 min 27 s]; (d) T2-weighted “hippocampal dedicated” coronal scan with 0.4 × 0.4 × 2.0 mm^3^ resolution, partial brain coverage and oblique orientation as described by Yushkevich et al. (2015).

Regarding whole-brain functional connectivity, connectomics changes after intervention will be investigated between pairs of regions of the whole brain (with particular interest in areas mainly involved in training-related cognitive processes) and in the global and local topological properties of large-scale networks through graph theoretical approach.

## 3 Discussion

There is an urgent need to develop population-level, low-cost interventions that will reduce the risk of cognitive decline and dementia. In this framework, we are currently carrying out the present RCT study to evaluate the effects of a novel immersive virtual reality and telemedicine-based multi-component intervention focused on specific cognitive functions (i.e., long-term associative memory, working memory binding, spatial pattern separation) and education program in individuals with subjective cognitive decline. In fact, it has been widely demonstrated that SCD is a condition at high risk for cognitive decline and clinical progression in dementia (Jessen et al., 2020).

The design of this study has many strengths with respect to previous interventions described in the literature. These strengths include the targeted cognitive training approach, the multi-component nature of the intervention, the use of immersive virtual reality, and the telemedicine method.

The first relevant point is the development of a cognitive training specifically designed and adapted to the cognitive difficulties of individuals with SCD. Recent findings suggest that self-perceived cognitive decline complained by SCD, but as yet undetectable using the traditional neuropsychological tests, actually reflects objective alterations affecting specific and more demanding processes (e.g., associative learning and recall, working memory binding, pattern separation processes; De Simone et al., 2023b; Kormas et al., 2020; Markova et al., 2023). Accordingly, one possible way to improve the efficacy of cognitive interventions in SCD, and thus produce more robust and broadly generalizable effects compared to previous results in the literature, may be to target specific cognitive processes, as those that have been found precociously and objectively compromise in SCD (e.g., associative memory for face-name associations) rather than more general cognitive functions (e.g., word list recall). To the best of our knowledge, only one study tested the efficacy of memory training that targets specific cognitive processes in SCD (i.e., memory for face-name associations) showing promising results (Pike et al., 2018). However, this finding is limited by some methodological shortcoming (e.g., single-session training, no RCT design) and thus more evidence is still needed.

The second point of strength is the multi-component nature of the intervention. As reported in the introduction, up to 40% of dementia cases could be prevented by acting on several modifiable risk factors, including lifestyle factors as cognitive activity, physical exercise and dietary habits (Livingston et al., 2020). The implementation of interventions targeting multiple dementia risk factors simultaneously can thus represent an effective strategy to increase efficacy. Health-related education and lifestyle interventions aimed at improving brain health and cognitive functioning before the onset of dementia have demonstrated overall positive effects on both subjective and objective cognitive function in healthy older people and in individuals with SCD (Sheng et al., 2020; Cohen-Mansfield et al., 2015). Thus, combining cognitive training with lifestyle modification programs could potentially result in more beneficial effects on cognitive, behavioral, and psychological functioning compared with interventions focusing only on a single component.

From a technological point of view, another point of strength is the implementation of the intervention in immersive virtual reality (IVR). A well-known obstacle in the field of cognitive interventions is the generally poor evidence for transfer effects of cognitive improvement to outcomes related to patients' daily living (Willis and Belleville, 2016). Another potential issue is a lack of motivation to participate in the assigned training, which is often affected by low pleasantness and attractiveness of the activities to be carried out. These problems probably contribute to attrition rates that are often high in training programmes (Willis and Belleville, 2016). IVR has been suggested as a feasible solution to accommodate these issues because it offers a more ecological environment for training activities that can simulate events that individuals might encounter in their daily lives, and with which they can interact in a naturalistic way by using their own bodies. This could result in a larger transfer effect (Tieri et al., 2018). Moreover, IVR has proven to induce the sense of presence (i.e., illusion to be in the virtual space which allows eliciting realistic reactions to virtual stimuli) and thereby increase engagement, motivation and the feeling of entertainment in users (Tieri et al., 2018; Sanchez-Vives and Slater, 2005; Fusco and Tieri, 2022); this could, in turn, result in reducing attrition rates in training programs. Despite the extended recent interest in the use of IVR for cognitive rehabilitation across several psychiatric and neurological disorders, including mild cognitive impairment and dementia (Jahn et al., 2021; Ren et al., 2024), this field is still unexplored in SCD. To the best of our knowledge, only one study to date has tested the efficacy of IVR cognitive training in SCD (Kang et al., 2021), reporting promising results.

A final point of note is the use of a telemedicine approach. Compared with traditional in-person interventions, in fact, the implementation of intervention that people can perform directly at their own homes by using a new generation of virtual reality HMD remotely controlled in real-time could represent an important step forward for distance treatment and a valuable contributor in improving the continuity of care. In fact, its cost-effectiveness, accessibility, flexibility, and comprehensiveness make it an ideal option for individuals unable to attend outpatient appointments due to various barriers such as work, distance, transportation issues, or disability. While recent reviews have demonstrated the effectiveness and feasibility of telerehabilitation for cognitive function in individuals with mild cognitive impairment and dementia (De Simone et al., 2023a; Cotelli et al., 2019; Costanzo et al., 2020), research on its application for subjective cognitive decline remains limited.

In the evaluation of the intervention and its possible effectiveness, some potential critical aspects and open issues should be also considered. The first regards technological expertise: it is more common for older individuals to show limited familiarity with the technology and may, therefore, have difficulties in using VR devices and managing the related software. However, we believe that the in-person training provided prior to the intervention, together with guaranteed technical support throughout its duration, could be useful for mitigating this negative effect.

Second, IVR has been related to cybersickness, a phenomenon often experienced with immersive VR environments. It typically manifests through adverse symptoms such as nausea, dizziness, headaches, and disorientation. Nevertheless, a recent systematic review of immersive VR studies conducted with elderly individuals indicated a minimal occurrence and low symptom ratings for cybersickness (Bauer and Andringa, 2020). Anyway, this aspect will be carefully examined through the analysis of data from the Simulator Sickness Questionnaire (Kennedy et al., 1993), as well as participants' qualitative reports, to thoroughly assess the intervention's usability and feasibility. Finally, and particularly relevant for at-home unsupervised interventions, is the issue of treatment adherence. Maintaining consistent engagement can be challenging outside of a controlled clinical environment. Our telemedicine approach, which includes scheduled virtual check-ins and remote monitoring functionalities, is specifically designed to explore strategies for supporting and improving adherence in this context. By examining participation rates, completion of assigned tasks, and qualitative feedback, our research aims to shed light on how remote support can influence long-term engagement and sustainability, addressing a critical open question in the management of these types of interventions.

In summary, the results of this randomized controlled trial will provide crucial evidence regarding the effectiveness of a novel multi-component intervention, integrating cognitive training and health education delivered via immersive virtual reality and telemedicine, for individuals at high risk of cognitive decline and dementia (SCD). Given its relatively low cost and accessibility, this approach has the potential to significantly contribute to primary prevention and early cognitive rehabilitation efforts aimed at reducing dementia risk.
